# Microbiome Research in Greece: A Comprehensive Bibliometric Study

**DOI:** 10.3390/microorganisms13040725

**Published:** 2025-03-24

**Authors:** Christos Stefanis, Christina Tsigalou, Ioanna Bezirtzoglou, Chrysoula (Chrysa) Voidarou, Elisavet Stavropoulou

**Affiliations:** 1 Laboratory of Hygiene and Environmental Protection, Department of Medicine, Democritus University of Thrace, 68100 Alexandroupolis, Greece; ctsigalo@med.duth.gr (C.T.); elisabeth.stavropoulou@gmail.com (E.S.); 2 School of Chemistry, University of Edinburgh, Scotland EH8 9YL, UK; bezirtzoglou.ioanna@gmail.com; 3 Department of Agriculture, School of Agriculture, University of Ioannina, 47100 Arta, Greece; xvoidarou@uoi.gr

**Keywords:** microbiome, gut health, microbiota, probiotics, brain–gut axis, intestine flora, immune system, bibliometric

## Abstract

Bibliometric analyses are increasingly used to evaluate scientific domains, revealing research trends, productivity, and impact. This study provides a bibliometric analysis of microbiome-related research conducted by Greek scientists. Data were retrieved from the Scopus database, using the keyword “microbiome” (English) for publications until December 2024. Bibliometric analysis was performed using VOSviewer and the bibliometrix package in R. Our findings indicate that research output has increased exponentially since 2018, with the National and Kapodistrian University of Athens and the Aristotle University of Thessaloniki leading microbiome research in Greece. Medicine, biochemistry, genetics, molecular biology, immunology, and microbiology are the predominant research fields. The keyword analysis highlights “microbiome”, “microbiota”, “probiotics”, “prebiotics”, “intestinal flora”, and “16S rRNA” as central topics. Additionally, we acknowledge the role played by alternative microbial markers, including 18S rRNA/ITS sequencing, for fungal diversity studies. This bibliometric study demonstrates a dynamic and evolving research landscape in Greece and highlights the international relevance of Greek contributions to microbiome science.

## 1. Introduction

### 1.1. Definitions of Microbiome

The word ‘microbiome’ is etymologically derived from two Greek words: ‘micros’ and ‘bios’. Early definitions comprised the elements of microbial communities and interactions of the formed biological networks. In the late 1980s, the definition evolved to include the habitat—a pre-defined environment—and the function of the communities at their core and the physiochemical properties of the microbiome [1,2]. It is important to highlight the addition of the spatial factor into the definition, combining the development of microbial communities within a three-dimensional ecological niche.

The most used definition was formulated in 2001, which describes microbial communities that include symbiotic and pathogenic microorganisms within an environment or a compartment in the body [3]. In addition to this new ecological dimension of the microbiome, other researchers have focused on the genetic and genomic dimension, the set of genes and gene expression in an environment with biotic and abiotic parameters [4].

Although various definitions of the microbiome have been proposed, they all had two main axes: the composition of the microbial communities in which microorganisms are present and the interactions between the environment and/or the host environment. The above emphasizes the interdisciplinary research perspective required when studying the microbiome’s concept and role [5].

While the human microbiome is well studied, gaps remain in understanding its ecological and functional relationships, and the research development in this field will continue to reveal new pathways regarding specific niches and interconnections that have not yet been fully characterized [6,7]. The phenomena of co-evolution of microorganisms with the human body also generate new research questions; as humans grow older, their metabolism changes and stressors are involved with a consequent variability in the composition of the microbiome. Finally, ecological questions about the relationships of the microbiome with the human body, as well as about the environmental relationships that develop within the microbiome, e.g., mutualistic relationships, prey–predator relationships, and symbiotic relationships and their interactions, have not yet been answered by the scientific community [5,6,7,8].

Table 1 presents some of the microbiome definitions with different conceptual contents. It is based on scientists’ efforts to answer questions concerning the composition of microbial communities, interactions within and outside microbial networks, interactions with the environment, spatial and temporal components, and questions based on the gene expression and evolution of these microbial populations through standard definitions.

### 1.2. Gut Microbiome and Health

The human microbiome has been linked to the immune system through complex mechanisms, systems, and networks. The contribution of the microbiome to a variety of functions such as digestion, vitamin production, homeostasis, intestinal barrier function, and angiogenesis in the human body has led to the conclusion that understanding the microbiome as an essential element of the human body will lead to a better understanding of immune behavior in a group of diseases [9]. A wealth of research implicates the gut microbiome and its homeostasis with complex chronic diseases such as inflammatory bowel disease (IBD), particularly Crohn’s disease and ulcerative colitis [10]. Although the role of the gut microbiota during IBD and the role of specific microorganisms in it (*Faecalibacterium*, *Subdoligranulum*, *Roseburia*, *Alistipes* sp.) has been partially elucidated and explained, the network of microbial, molecular, biochemical, and immunological interactions in the initiation and progression of these diseases has not been discovered or researched extensively [11,12,13]. The gut microbiota also plays a crucial role in scientific research on obesity, as demonstrated by a 2018 bibliometric analysis [14].

The composition of the gut microbiota plays an essential role in the onset and progression of obesity through the modification of metabolism, host homeostasis, and the way fat is stored in adipocytes [15,16]. Modern lifestyle and the use of antibiotics in infancy also affect the formation of the intestinal microbiome from birth, contributing to the development of obesity, which in turn causes autonomously and in combination additional health problems, such as coronary heart disease, type 2 diabetes, and an escalated incidence of various forms of cancer [17,18,19].

In addition to pathogenic conditions, the intestinal microbiome has also been associated with the longevity of elderly and significantly older adults. As has been emphasized above, the variability of the composition of the microbial communities of the intestinal tract underlines their significance in human health throughout their lives. The microbial and genomic architecture of the intestinal microbiome changes during ageing, as has been found in studies in different age groups. A decrease in its taxonomic diversity is directly related to it. The direct relationship between longevity and the gut microbiota of older adults is a significant research question, and the role of the gut microbiota in the human ageing process is expected to be proven in large-scale clinical studies and its association with other factors, such as probiotics [20,21].

The gut microbiota has attracted the research interest of scientists due to its complex and ecologically dynamic community that changes throughout the human lifespan and affects the development, maturation, and regulation of the immune system [22,23]. Numerous immunological and/or inflammatory diseases are related to the balance of the gut microbiota and the nervous system through the chemical and biological communication of the gut with the brain (gut–brain axis). Hence, the biochemical behavior of the brain can be influenced by the biochemical behavior of the intestinal microbiome, independently of the autonomic nervous system [24,25].

In this context, some studies show that changes in the intestinal microbiome can also affect human mental health. Communication between the gut and the brain through immunological, metabolomic, and endocrine pathways also affects various mental disorders such as depression and Parkinson’s disease. Changes in the intestinal microbiome in animal models and humans have been associated with a positive outcome for mental health-related diseases. This correlation has been observed in depressed individuals, who showed improvement when given therapeutic regimens with probiotic microorganisms, e.g., *Bifidobacteria* sp. [26,27]. Through neurotransmitters and hormones, which play a critical role in serotonin’s metabolism, the gut microbiota–brain axis is also linked with anxiety and behavior disorders [9].

Alterations in the gut microbiota have also been associated with other diseases and disorders, such as developmental disorders, autism spectrum disorders, and Rett syndrome [28]. In addition, reduced numbers of specific gut microorganisms such as *Bifidobacteria* and increased populations of *Clostridium* spp., *Desulfovibrio* spp., *Sutterella* spp., and *Veillonellaceae* sp. have been observed in individuals with autism spectrum disorders [29]. Furthermore, imbalances in the *Firmicutes*/*Bacteroidetes* population and ratio and high *Candida* populations have been reported in autistic individuals [30]. In conclusion, the modulation of the gut microbiome and the importance of the role and chemical intercommunication of the gut microbiome–brain axis may open up new therapeutic avenues in mental diseases and disorders [9].

Using bibliographic analysis and evaluating research through bibliometric indicators offers several advantages, such as the numerical documentation of research production and its influence and impact. Moreover, bibliometrics captures the availability and direction of funding sources in various research channels, highlighting all forms of collaboration from the level of authors to the level of countries. It encapsulates, inter alia, the assessment of research production in a field, the structure of research and science, the detection of contemporary and topical issues, and methods for developing research production with the help of quantitative and qualitative bibliometric indicators [31,32,33,34,35].

The present investigation aims to bibliometrically assess the scholarly output generated by Greek researchers on the microbiome. Initially, the annual volume of publications will be documented to illustrate Greek research activity. Furthermore, descriptive metrics will be employed for the articles that serve as the foundation for constructing bibliometric maps. Concurrently, the most impactful scientific publications, prolific researchers, and Greek academic institutions concentrating their research on the microbiome will be catalogued. Additionally, the scientific domains most closely associated with microbiome research will be emphasized. By generating bibliometric maps using the bibliometrics package of R, the Greek scientific community’s research trajectories and focal points engaged in microbiome studies will be elucidated, along with the temporal progression of Greek scholarly research in this field.

To summarize our research objectives, the current study will attempt to unveil the evolution of Greek scientists’ research activity over the past decades based on a comprehensive set of specific bibliometric indicators. We will also explore the universities and institutions that contribute to this field of microbiome research. Lastly, we will delve into the dominant themes and research pools in microbiome studies within the Greek scientific community, providing a comprehensive overview of the field.

## 2. Materials and Methods

**Figure 1:**
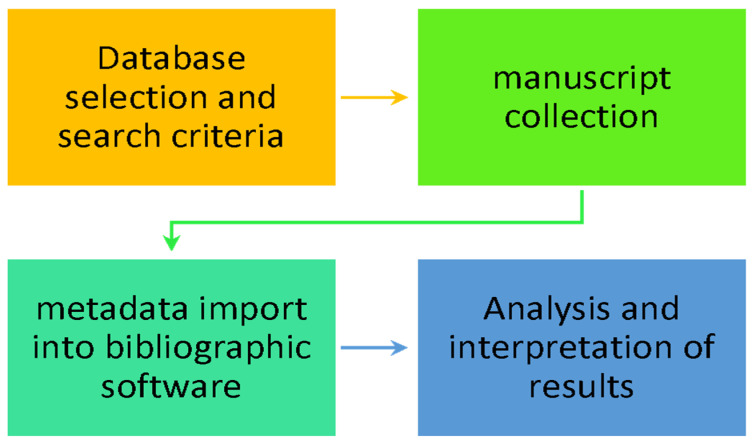
Research flow diagram.

The steps of this research are presented in the image below (Figure 1) and include the selection of the database, Scopus, the combination of appropriate keywords, and the collection of papers. Subsequently, a check is carried out for the accessibility of the selected documents and the correctness of the search results, as well as the removal of any duplicates or records that are the same in content but have a different document type. Finally, the results are exported in BibTeX format and imported into the R package, bibliometrix, and the corresponding web application biblioshiny. Conclusions regarding microbiome research within the Greek scientific community will be drawn. The Scopus database was selected to search for scientific papers related to the microbiome. This database is considered one of the leading databases for bibliography searches and conducting complex research, review studies, and meta-analyses [36]. In addition, it has a wide range of search options based on various criteria, e.g., year, document type, language, publication stage, scientific area, accessibility, etc. It also has an easy-to-use menu for using search operators and the option to export bibliographic metadata. Finally, the Scopus database includes documents from 7000 research publishers and over 2500 titles of scientific journals [37,38,39].

**Figure 2:**
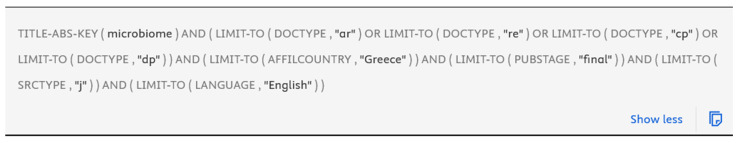
Scopus database search criteria and filters.

After various combinations of keywords and search operators, the word “microbiome” was selected in English to include and compare the annual production of records up to and including the end of 2024. The search was performed in the fields “Article title, Abstract, Keywords” by selecting the corresponding option from the Scopus search filter options menu. The country “Greece” was also selected, which resulted in the retrieval of documents from Greece and Greek researchers publishing in collaboration with universities abroad. In addition, the types of chosen documents were scientific articles, literature review articles, and articles from conference proceedings, “articles-conference paper-review”, and the language “English” (Figure 2).

The above search was conducted in December 2024 and generated 720 records. All works were saved in BibTeX (.bib), RIS, and Excel (.csv) format with all bibliographic metadata. The bibliometric analysis was performed using the bibliometrix package in R, a widely used tool for science mapping and data visualization [40]. After installing R, we then installed R Studio 2024.09.01, the bibliometrix library (install.packages (“bibliometrix”, dependencies = TRUE), library(bibliometrix), and used the biblioshiny web application.

A bibliometric analysis was conducted using a structured methodology that systematically integrated data preprocessing, network analysis, and clustering techniques to analyze the bibliographic metadata. This streamlined approach follows the established bibliometric research guidelines to ensure a straightforward and transparent assessment. The dataset was sourced from Scopus and selected to comprehensively cover the domain under study. Before analysis, the dataset was pre-processed to enhance accuracy and consistency. Duplicate removal, ensuring that redundant entries were eliminated to prevent overrepresenting specific studies, author names, and keywords were standardized, and variations in spelling and formatting were addressed to create uniformity across the dataset. Consequently, filtering based on minimum occurrence thresholds retained only terms appearing at least 40 times to maintain statistical relevance and focus on key thematic trends. For keyword network mapping and co-occurrence analysis, VOSviewer 1.6.20 visualized relationships between key terms. The association strength normalization method balanced term distribution and accurately depicted conceptual linkages within the field. Clustering analysis was performed using the Louvain algorithm to detect structural patterns in the data, which is widely recognized for its efficiency in identifying modular structures. The clustering resolution parameter was fine-tuned to achieve an optimal balance between thematic granularity and interpretability, ensuring that meaningful research clusters were effectively identified [41,42,43].

## 3. Results

**Figure 3:**
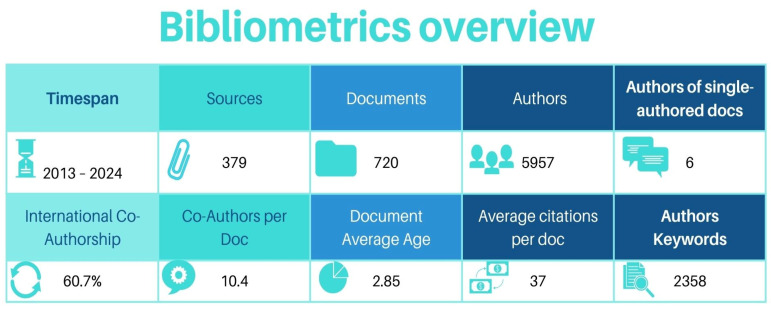
Main bibliometrics information.

Figure 3 includes statistics on the production of scientific publications, author collaboration, and publication impact through the number of citations and provides a general overview of the primary information extracted from the bibliographic metadata.

The total number of keywords from the keywords plus system (ID) is 7333. The author’s keywords defined equals 2358, and the total number of authors is 5957. Notably, only six authors have published monographs. The average number of authors per manuscript is 10.4, highlighting powerful scientific collaboration. Furthermore, the percentage of international collaborations aggregates to 60.73%, demonstrating the global dimension of scientific activity. The average number of citations per publication amounted to 34.64, identifying a high scientific impact. More generally, the data indicate a highly collaborative and internationalized research activity, with many citations per publication and the prevalence of collaborative research projects, as shown by the small number of monographs.

**Figure 4:**
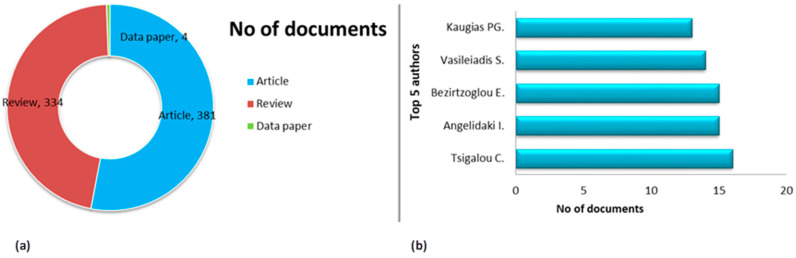
(**a**) Document types; (**b**) top authors.

Figure 4 represents the total number of documents retrieved based on the search criteria and the five most productive authors on the microbiome. Almost all the documents concern scientific articles and review articles, demonstrating that the Greek scientific community systematically reviews the existing literature and produces articles that collect research data to promote existing knowledge and new developments. Furthermore, scientific articles are the most numerous of all document types, demonstrating the experimental interest of Greek researchers in all scientific directions of microbiome research.

Based on their research output, the five most productive authors come from Greece and abroad. Three of the five most productive are women. The total number of manuscripts of the five top Greek researchers ranges from 16 to 13.

The most productive author, Tsigalou Christina (https://www.scopus.com/authid/detail.uri?authorId=16647931600, accessed on 31 January 2025), from the Democritus University of Thrace, has a h-index of 23. Moreover, the second, Angelidaki Irini (www.scopus.com/authid/detail.uri?authorId=6603674728, accessed on 31 January 2025), has scientific affiliations with research institutions abroad, specifically in Denmark, with a h-index of 107 and 43.981 citations. The remaining three authors come from institutions in Greece, Bezirtzoglou Eugenia (www.scopus.com/authid/detail.uri?authorId=7003748111, accessed on 31 January 2025), with a h-index of 37, Vasileiadis, Sotirios (www.scopus.com/authid/detail.uri?authorId=54080600200, accessed on 31 January 2025) from the University of Thessaly, with a h-index of 26, and Kougias, Panagiotis G (www.scopus.com/authid/detail.uri?authorId=36548398300, accessed on 31 January 2025), with a h-index of 44, from the Hellenic Agricultural Organization DIMITRA. 

In summary, the authors identified in this bibliographic survey on the microbiome are prolific and have much recognition and have published many scientific works. They are scientifically active in various fields, integrating microbiome research into multiple applications in medicine, environmental microbiology, ecology, hygiene, immunology, microbial ecology, waste management, biotechnology, molecular biology, and pharmacology.

**Figure 5:**
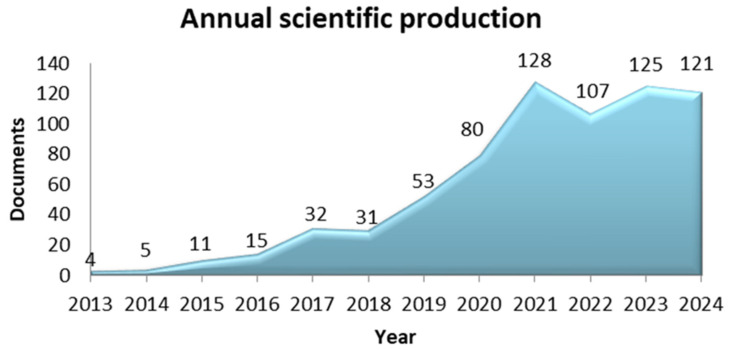
Annual scientific production of manuscripts.

Figure 5 depicts the annual research production of scientific articles among the Greek research community. The year in which the word “microbiome” was first recorded was 2013. The significant production of scientific documents began after 2017 and increased significantly, almost exponentially, after 2018. Notably, in the last four years of the search, 2021–2024, the annual production of articles exceeded 100 per year. Overall, the trend of manuscript publications shows substantial growth in scientific output over the search period, with a marked peak in 2021 (128 records), followed by stabilization at a high level of research production.

**Figure 6:**
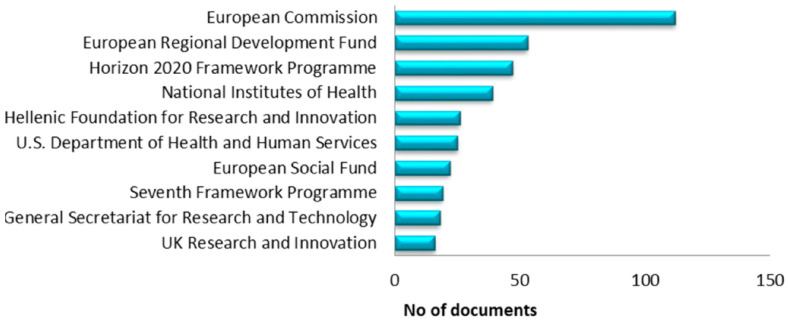
Documents per funding sponsor.

**Figure 7:**
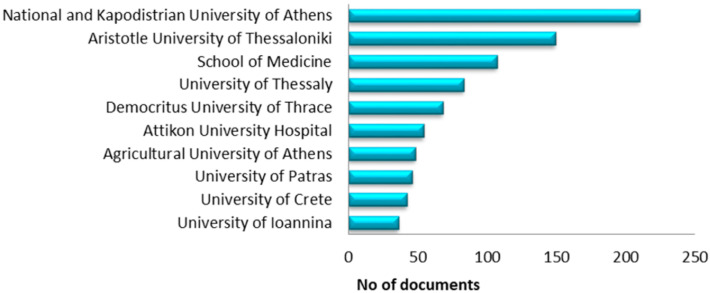
Documents per affiliation.

Figure 6 and Figure 7 illustrate the sources of funding for research related to the microbiome and the top ten universities in terms of article production. The European Union, with various funding mechanisms and schemes, dominates the field of research funding, and the Greek research community often turns to European funding sources (253 manuscripts in total). The first Greek institution, the National Institutes of Health, ranked fourth (39 manuscripts). In contrast, two additional institutions emerged from Greece, which were listed in sixth and ninth positions: the Hellenic Foundation for Research and Innovation (26 manuscripts) and the General Secretariat for Research and Technology (18 manuscripts).

The scientific institutions with the highest production of scientific articles are the National and Kapodistrian University of Athens (NKUA) with 210 and the Aristotle University of Thessaloniki (AUTH) with 149. The top five institutions include the School of Medicine, the University of Thessaly, and the Democritus University of Thrace (DUTH), with 107, 83, and 68 articles, respectively. Interestingly, one hospital also shows a significant research output, the Atticon University Hospital, with 54 published studies about the microbiome. In conclusion, half of the most productive institutions in microbiome research are peripheral universities, and the remaining three belong to the two largest cities in Greece.

**Figure 8:**
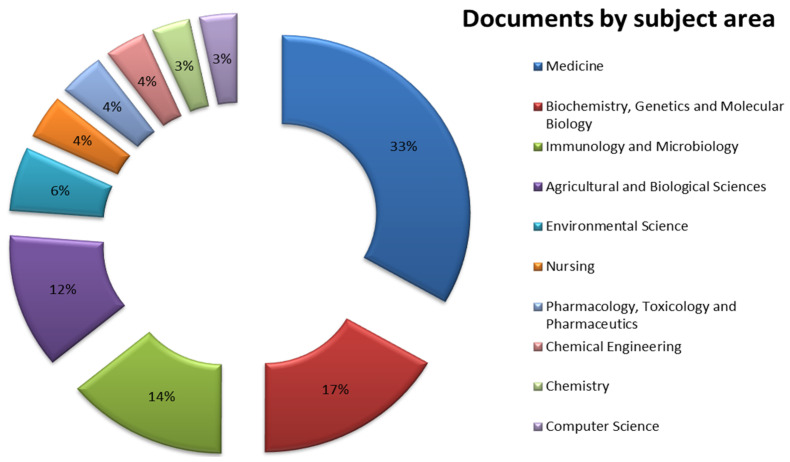
Documents per subject area.

Figure 8 presents the percentage distribution of articles in this research according to the subject area, showing the ten regions that hosted the most documents. The scientific areas that appear to be most related to the microbiome include, among others, medicine (33%), biochemistry, genetics, and molecular biology (17%), and immunology and microbiology (14%), indicating an intense research focus on medical and genetic sciences as well as in infectious diseases and microbial research. In addition, agricultural and biological sciences (12%) and environmental science (6%) focus on food and crop sciences, climate change, ecosystems, and sustainability. Lastly, the scientific areas and disciplines of nursing, pharmacology, toxicology, and chemical engineering appear to be related to microbiome research and innovations, with their percentages below 5%.

**Figure 9:**
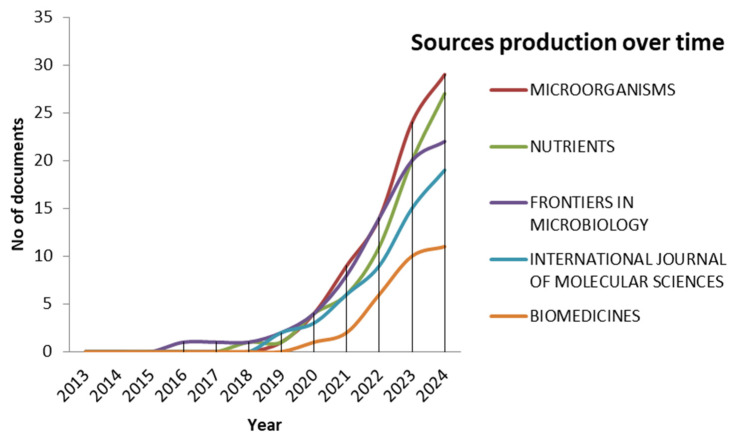
Sources production over time.

Figure 9 represents the cumulative increase in publications from the top five scientific journals regarding microbiome research from 2013 to 2024. Each line represents a different journal, illustrating the cumulative number of publications annually.

We observe three phases of the rise in the production of evidence during the research period. Initially, and until 2018, there was slow activity. *Frontiers in Microbiology* began a slight upward trend, specifically after 2015. The next phase is distinguished by the production of articles between 2018 and 2020, where the production of articles begins to rise significantly. All journals increased their publications. The third phase, between 2020 and 2024, presents a pattern of significant increase in all journals. *Microorganisms* and *Nutrients* were the most popular publications dealing with the microbiome, while *Biomedicines* followed a more gradual trajectory.

Meanwhile, *Frontiers in Microbiology* and the *International Journal of Molecular Sciences* maintained steady growth. This recent pattern confirms a boost in research activity that implies a higher volume of publications. The COVID-19 pandemic may have fueled this increased published research addressing microbiology and health sciences topics.

Table 2 presents the five most influential articles on microbiome research published between 2015 and 2020.

The first and most influential article highlights the potential role and relationship between *H. pylori* and the microflora of the upper and lower digestive tract, as well as the possible role of microbiome composition in the long-term effort to eliminate and treat this microorganism [44]. Based on total citations, the next most cited article was published in 2020 and reviews the definition of the latest technological and scientific developments in microbiome research [6]. The third article examines potential vital factors, such as altered gut microbiome and mechanistic factors in obesity and cancer. The manuscript investigates the interaction between key potential mechanisms and risk factors that affect target tissues, leading to their reconstruction into a cancerous phenotype [45]. The fourth most cited article highlights the spread of antibiotic-resistant strains from farmed animals and the potential expansion and proliferation of such strains in the environment [46]. The last article on the list of the most influential articles examines the bidirectional relationship between the gut microflora and the brain and how this relationship contributes to the pathogenesis of certain disorders involving brain inflammation, the immune system, and the nervous system [47].

**Figure 10:**
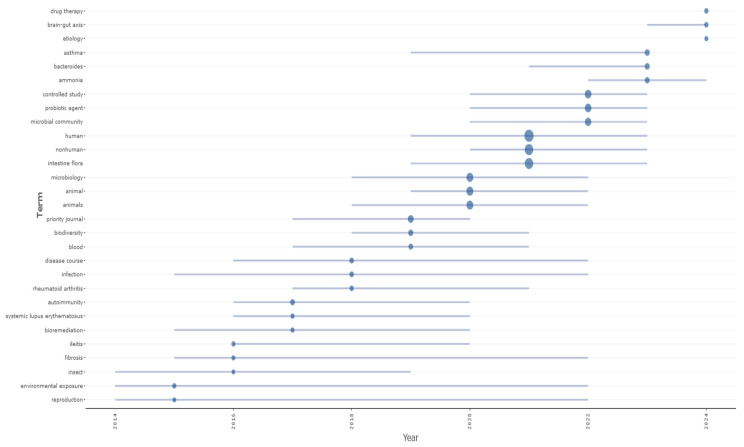
Trend topics of keywords plus (a blue bubble depicts every topic; the bubble size is proportional to keywords plus occurrences—minimum word frequency = 5. The grey bar illustrates the 1st and 3rd quartiles of the occurrence distribution).

Figure 10 represents the most trending topics, based on the frequency of word occurrences, in the field ‘keywords plus’. The entire search time is included, and the minimum occurrence value of a word is set to 5. These keywords are automatically generated and emerge from a manuscript’s citation list. Visualizing and detecting them has become popular in bibliometric analyses because they provide a detailed picture of a topic’s research depth and scientific content [48].

The horizontal axis shows the temporal aspect, while the vertical axis shows the keywords. “Microbial community, intestine flora, probiotics, bacteria, microbiology” are terms related to studying the microbial community, the intestinal flora, and their relationship with health. Their increasing presence in recent decades reflects the growing interest in the role of the microbiome in human physiology and pathology.

Keywords plus like “Drug therapy, controlled study” indicate the increasing effort to determine the effects of drug therapies, especially regarding the microbiome and chronic diseases. The appearance of “asthma, infection, blood, disease course, autoimmune, rheumatoid arthritis, systemic lupus erythematosus” reflects the scientific community’s focus on diseases related to the immune system and inflammation. The term “infection” appears relatively early, representing the long-standing interest in infectious diseases. Terms like “autoimmune, rheumatoid arthritis” signal the increased interest in autoimmune diseases. Moreover, “ecology, biodiversity, environmental exposure, reproduction” expresses the association between organisms and the environment and their impact on human health. The keywords “ammonia, bioremediation, diet, fibrosis” demonstrate research directions on metabolism, nutrition, and bioremediation methods. The term “diet” has emerged in recent years, which may be related to the contemporary focus on the role of diet and nutrition in regulating the microbiome and human health. Lastly, the escalated research around the brain–gut axis (“brain–gut axis”) revealed the link between the central nervous system and the gut microbiome and the potential implications for neurological and psychiatric health.

**Figure 11:**
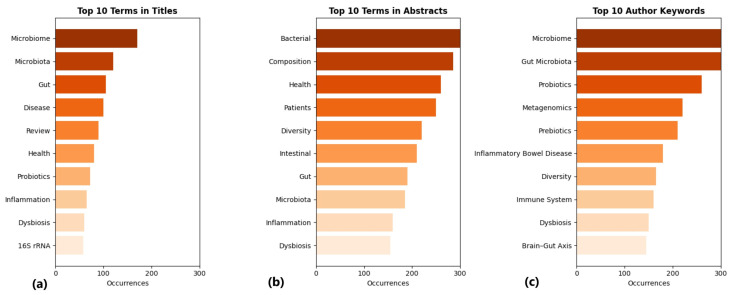
Frequency bar charts of the (**a**) title; (**b**) abstract; (**c**) keywords (10 words).

The subsequent figure (Figure 11) outlines the three bar charts of the 10 most frequently occurring terms in the collected documents’ Title, Abstract, and Keywords Sections.

The bar charts above illustrate that the most frequent keywords appeared more than ten times in scientific titles, abstracts, and keywords, highlighting key themes in microbiome research. The most frequent words in titles include “microbiome”, “microbiota”, “gut”, “disease”, and “review”, indicating that most research focuses on the microbiome’s relationship with diseases and systematic reviews on the topic.

Abstracts use a broader range of terms, such as “bacterial”, “composition”, “health”, “patients”, “diversity”, and “intestinal”. This suggests that the studies emphasize the composition and biodiversity of the microbiome, as well as its role in human health. In the Keywords Section, the most dominant word is “microbiome”, followed by “gut microbiota”, “probiotics”, “metagenomics”, “prebiotics”, and “inflammatory bowel disease”. This specifies the significance of probiotics and metagenomics in microbiome research and their relationship to chronic intestinal diseases.

Overall, the term “microbiome” is the most dominant in two of the three fields. The Titles focus on general topics such as reviews and their relationship with disease. The Abstracts include more terms related to the composition, diversity, and impact on human health. The keywords centered on the genetic analysis of the microbiome and the use of probiotics and prebiotics in the study of the gut microbiome.

Network mapping was used to analyze microbiome research’s conceptual content, dynamics, and structure. This method detects the co-occurrence of the most frequently used words in the body of literature identified by bibliometric analysis, namely the sum of scientific articles and studies (co-occurrence analysis).

Briefly, this analysis is based on the fact that terms that tend to appear together will have a greater thematic and conceptual relationship. The aim is to construct bibliometric maps that graphically display a field’s research horizons, conceptual thematics, and the research connections between scientific fields. In this bibliometric analysis, the maximum number of occurrences on the map of the 55 most frequently occurring words was defined, setting the minimum number of occurrences to 40, with a normalization criterion (normalization) of the relationship between the terms (association) and a Louvain clustering algorithm. Larger nodes correspond proportionally to more frequently used keywords.

This algorithm remains quite popular for detecting structures in a bibliometric network and is very effective for detecting subgroups within large networks. The Louvain algorithm is also applied to large networks, reducing the computational complexity required and making it particularly efficient in co-occurrence networks for understanding research trends and their structures. It is now an analysis option in all bibliometric software, with various additional parameterization options [49,50].

**Figure 12:**
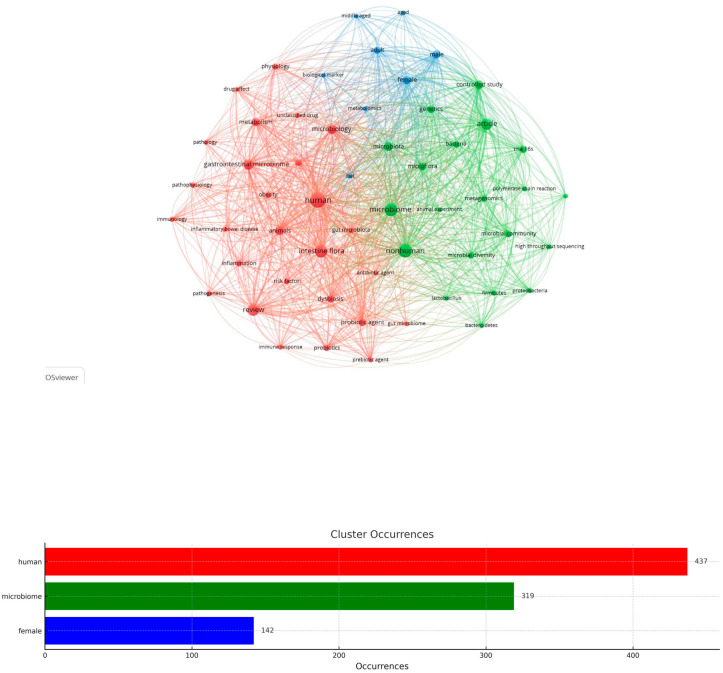
Keyword co-occurrence analysis and cluster distribution (55 words).

Figure 12 shows the co-occurrence network of keywords as generated by VOSviewer 1.6.20. Illustratively, each cluster is depicted in a different color. Each term belonging to one cluster cannot belong to another. Also, the size of the lines connecting terms depends on the number of co-occurrences of the terms they connect. It shows the intensity of the relationship between them (the thicker the line, the stronger the occurrence).

The bibliographic network is divided into distinct clusters, with each color representing a different topic area. The red cluster (Biomedical and Health-Related Topics) includes terms such as human, gastrointestinal microbiome, obesity, inflammation, review, metabolism, and immunology, suggesting topics related to human health and gut microbiota. The green cluster (Microbiology and Genetics-Related Topics) includes keywords such as microbiome, microbial diversity, metagenomics, bacteria, and 16S RNA, indicating studies on microbial taxonomy and sequencing techniques. The blue cluster (Demographics and Study Design) includes keywords such as male, female, elderly, adult, and controlled study, representing demographic information and study methodologies. The term “human” appears at the center of the network, documenting the great value of human microbiome research. Other key terms such as microbiome, gut flora, and dysbiosis indicate that research primarily focuses on microbial communities in human health and disease.

When interpreting the bibliometric network, strong linkages between keywords related to microbiology, genomics, and medical sciences are revealed, indicating the high degree of interdisciplinary research and collaboration of Greek researchers.

This bibliographic network shows that research on the microbiome is interdisciplinary, covering areas such as medicine and biology (red cluster), microbiology and genomics (green cluster), and demographic and clinical studies (blue cluster). The study of the microbiome is one of the most dynamic research fields, with particular emphasis on metagenomics, clinical application, and impact on public health. At the same time, the bibliographic network illustrates the structure and main thematic directions of research on the microbiome. The strong connections between keywords highlight the field’s complexity and evolution towards understanding the interaction between microorganisms, the human body, and the environment.

Table 3 lists the keywords in three thematic clusters, corresponding to different scientific approaches to studying the microbiome and related biological parameters, as illustrated in Figure 12.

The red cluster includes keywords related to the human microbiome, metabolic disorders, pathogenesis, and immune responses. The main thematic sections of the red group include the human microbiome and intestinal flora (“human, intestine flora, gut microbiota, gut microbiome, gastrointestinal microbiome, microbiology”), with a focus on the function and structure of the microbiome and its multifactorial role in the human intestine. Also, through terms such as “inflammation, inflammatory bowel disease (IBD), immune response, immunology, pathogenesis, pathology, pathophysiology, and risk factor”, the red cluster includes research on the relationship of the microbiome with chronic inflammatory diseases such as IBD, and various immune responses. In addition, this cluster highlights the metabolic processes and the dysbiosis (“metabolism, dysbiosis, obesity, probiotic agent, probiotics, prebiotic agent”), namely the study of the disruption of the composition of the microbial flora and its association with metabolic diseases such as obesity. The analysis of the effects of drugs and therapeutic approaches such as antibiotics on the evolution and formation of the intestinal microbial flora is highlighted by the terms “antibiotic agent, drug effect, and unclassified drug”.

In conclusion, the red cluster and its keywords are mainly related to studying the human microbiome and its role in inflammatory disorders and metabolism. Emphasis is placed on pathological conditions, immune responses, and possible treatments with probiotics or drugs.

The green cluster focuses on microbiological analysis, genetic identification, and methods for studying the microbiome. Specifically, the terms “microbiome, microflora, microbiota, microbial community, microbial diversity, bacteria, Firmicutes, Proteobacteria, Bacteroidetes, and lactobacillus” show the research effort to systematically understand, classify and study the diversity of microbial communities and their composition. The immediately following terms in this group also delve into the methods for their elucidation, namely with genomic and laboratory techniques and methods, which focus on sequencing and molecular biology technologies, such as metagenomics and 16S RNA sequencing (“metagenomics, high-throughput sequencing, RNA 16s, polymerase chain reaction, DNA extraction”).

Lastly, understanding the function and composition of the microbiome requires studies in the first phase with animal models and the design of complex experiments (“nonhuman, controlled study, article, animal experiment, and genetics”). The green cluster emphasizes genetic techniques and advanced technologies, such as metagenomics, which are applied to the study of the composition of the microbiome to allow for their genetic recognition and identification.

The third cluster of the bibliographic network, the blue one, comprises keywords related to human demography, study methodology, and biological metabolic analyses. In particular, the demographic data of the research mainly concern the study of the evolution of the microbiome during a lifetime, as the human organism matures and ages and its differentiation based on gender, as can be seen from the terms female, male, adult, middle-aged, and aged.

Greek scientists’ research attempts are also concerned with the contribution and discovery of new biological indicators and metabolism (biological markers, diet, metabolomics) to decode the microbiome with biological indicators and dietary habits and patterns. In conclusion, the blue group thematically combines the study of demographic factors in the microbiome and the metabolic analysis of microorganisms through biological indicators.

**Figure 13:**
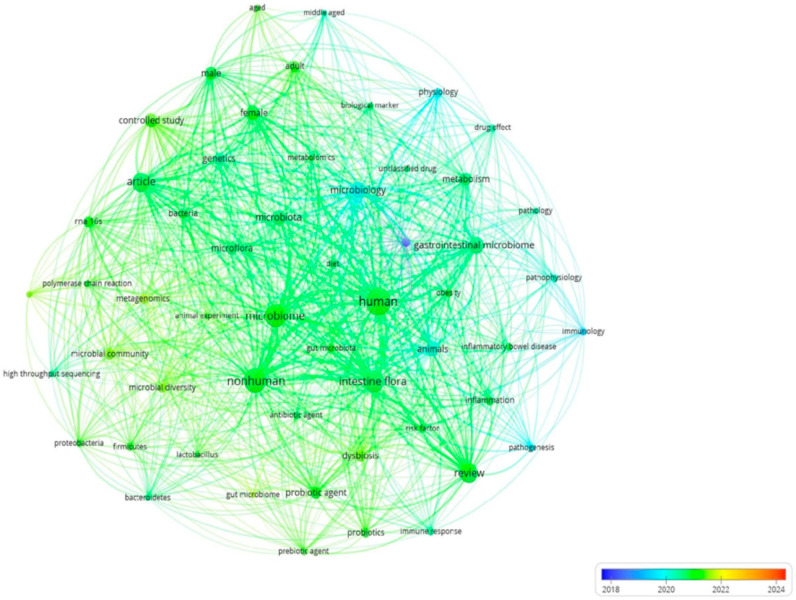
Timeline of microbiome research in Greece (2013–2024).

Figure 13 presents a keyword co-occurrence network generated by VOSviewer 1.6.20, with a time scale that highlights the evolution of microbiome research by the Greek academic community. This visualization is beneficial for mapping research trends, identifying dominant topics, and monitoring the most recent foci of research interest.

The timeline of microbiome research, illustrated through different colors in the bibliographic network, highlights how scientific interests have evolved, shifting from broad biological studies to more specialized, applied research focused on the microbiome.

The early phase of research between 2018 and 2019, shown in blue, included terms such as “human”, “microbiota”, “intestinal flora”, “bacteria”, and “microbiology”, which shows that the research was trying to decipher the microbiome through fundamental biological principles and descriptions of the microbial flora. Subsequently and until 2022, terms such as “immune response”, “probiotics”, “dysbiosis”, “gut microbiota”, and “inflammatory bowel disease” are identified, indicating a shift in the Greek academic community towards connecting research on the microbiome with the gastrointestinal system and its role in specific intestinal diseases.

The most recent research trends, highlighted in yellow and orange, covering the year 2024, show the entry of new research protocols and the attempt to document the composition of the microbiome by applying advanced genomic techniques, as well as through the development of clinical indicators (“high-throughput sequencing”, “metagenomics”, “biological marker”, and “controlled study”), showing that research is now focused on the application of advanced genomic techniques and clinical indicators. Finally, the term “diet” strongly links microbiome research and its connection with nutrition.

In conclusion, the bibliographic review shows a shift from the general description of the microbiome to more specialized clinical and genetic studies. The Greek scientific community is moving from the phase of mapping the microbiota to the phase of understanding its function to find therapeutic applications. Consequently, studies are being planned on the role of the microbiome in autoimmune, metabolic, and intestinal diseases. Overall, microbial communities’ analysis and identity, comprising the microbiome, are increasingly based on advanced genetic techniques, genomic approaches, and omics technologies.

## 4. Discussion

The bibliometric analysis performed through keyword co-occurrence networks, trend topics, timeline network, bibliometric indicators, and word clouds allows us to map the research fields and identify the main topics studied by Greek scientists. We then used these results to select articles substantiating or reinforcing the same research patterns. In the red cluster, the thematic focus refers to the microbiome’s relationship with diseases (metabolic, neurological, and autoimmune), its executive role in therapeutic approaches and immunology, and the temporal transition from general analyses to more specialized treatments.

Greek scientists’ research on the microbiome has shown significant progress in recent years, especially in areas related to the gut microbiota, probiotics, and their respective clinical applications. The Greek research output may be smaller than global players such as the United States, China, and the United Kingdom. Still, it remains collaborative due to the rate of co-authorship in publications. Several studies by Greek researchers are also in line with international research trends, with a high degree of interdisciplinary research, such as the diversity of the human microbiome, its impact on metabolic disorders, computational biology, bioinformatics, and research on the gut–brain axis [51,52]. The gut–brain axis, particularly, has been highlighted in understanding neurological disorders due to dysbiosis [52]. This aligns with global efforts to map the microbiome’s influence on neurodegenerative diseases. It should also be noted that many Greek studies are still at an exploratory stage and, in the future, will also be expanded to precision medicine applications concerning the human microbiome.

The Greek research community is quite active and productive in scientific areas related to microbiome research. For example, Greece ranks 11th among Western European countries in medicine–microbiology (medical) and 15th in gastroenterology, with an h-index of 153 and 141, respectively. The h-index for biochemistry, genetics, and molecular biology is 153, and Greece comes 11th among 28 Western European countries. In the same vein, the percentage regarding openness was almost 68% in 2023, while one in two documents are registered as open access from 2015 onwards. Moreover, Greek researchers’ openness, outreach, and extroversion as an indicator of international cooperation reached 56% in 2023. This percentage has been moving well above 50% since 2016 in medicine [53,54].

Investment and research strategies are crucial for developing microbiome research, considering its policy and funding implications. Our findings on funding flows show a mono-thematic focus from European funding sources. In contrast, national sources such as sequencing platforms and computational modeling facilities are limited in supporting large-scale research. At the international level, funding structures directly influence microbiome research’s development and translational potential [55].

The analysis of the articles in the red cluster confirms that the gut microbiome plays a crucial role in the pathogenesis and progression of various diseases, such as neurodegenerative disorders, hypertension, autism, cancer, inflammatory bowel diseases, and epilepsy. The majority of studies have highlighted dysbiosis as a central pathophysiological mechanism, suggesting that imbalances in the ratio of the bacterial phyla Firmicutes/Bacteroidetes are associated with neurodegenerative diseases (e.g., Parkinson’s and Alzheimer’s disease) [56], cardiovascular diseases and hypertension [57], and cancer [58].

In addition, particular emphasis is placed on the gut–brain axis, where the microbiome appears to influence neurotransmission, inflammation, and intestinal barrier permeability, shaping and determining many factors that affect neurological health [59]. These studies report that the intestinal microbiota can influence the synthesis and release of neurotransmitters, such as dopamine and serotonin, directly affecting behavior, cognitive function, and psychiatric disorders. These data confirm that the microbiome is now a key target in research on neurological and metabolic diseases.

Furthermore, research by Greek scientists detected by this bibliometric analysis highlights the role of the microbiome as a therapeutic target, either through antibiotics (such as rifaximin in epilepsy [60]), or through probiotic and nutritional interventions. These results show that probiotics and prebiotics can regulate inflammation and reduce intestinal dysbiosis, thus offering alternative therapeutic approaches for chronic diseases. A systematic literature review on hypertension revealed that low microbial diversity is associated with high blood pressure. Concurrently, microbiome metabolites (e.g., SCFAs) may have a protective effect [61,62]. Furthermore, microbiome studies in oncology patients have shown that the intestinal microbiota influences estrogen metabolism and immune regulation, making it an essential factor in the development and treatment of cancer [63]. Overall, the research output highlights the transformation of the microbiome from a biological marker to a potential therapeutic target, reinforcing the need for further clinical trials and multivariate studies that will allow for its full utilization in personalized medicine.

Research by Greek scientists aims to demonstrate the central role of the gut microbiome in metabolic diseases, such as obesity, type 1 diabetes (T1D), and chronic inflammation, linking dysbiosis to alterations in metabolism and immune responses [64]. Specific *Bifidobacterium* species and metabolites such as SCFAs (short-chain fatty acids) lead in regulating intestinal inflammation and the pathogenesis of obesity and type 1 diabetes. The researchers found that obese individuals had increased levels of *B. longum subs. infantis* and *B. breve*, while those with T1D showed metabolic disorders and variations in the profile of Bifidobacterium species, indicating that changes in the microbiome are directly linked to metabolic pathologies [65].

Another study demonstrated the effect of probiotics on glycemic control in children and adolescents with type 1 diabetes. Interestingly, although probiotic interventions have been considered protective, the meta-analysis recorded worse glycemic control and increased glucose and HbA1c values in patients receiving probiotics. These data suggest that the effect of probiotics depends on the host microbial profile, strain selection, and metabolic interactions with the immune system [66]. Furthermore, another study shows how dysbiosis is associated with the increased production of lipopolysaccharides (LPSs), which activate inflammatory pathways and contribute to the pathogenesis of diabetes and obesity [67]. Regarding obesity and its systemic effects on the microbiome, it is recorded from the bibliography of Greek researchers that obesity affects the composition of the microflora not only in the intestine but also in the liver and lungs, modifying and altering the ratio of *Firmicutes* and *Proteobacteria*. This microbiome connection with other organs highlights its role in developing systemic inflammatory responses associated with metabolic and autoimmune diseases [67].

Furthermore, the potential contribution of antibiotic supplementation in the predisposition to obesity underlined that this specific exposure during infancy is linked with alterations in microbial richness and an increased risk of obesity in adulthood [68]. The loss of specific bacterial strains, such as Lactobacillus, due to antibiotic exposure affects energy metabolism, leading to increased fat deposition. Finally, another study argues that chronic inflammation (low-grade inflammation) is a key link between the microbiome and obesity. The metabolic effects of the microbiome are not only limited to weight gain but also affect immune homeostasis, enhancing the production of pro-inflammatory cytokines [69]. This relationship between intestinal microbiota and chronic inflammation makes the microbiome a key therapeutic target for treating obesity and metabolic disorders through personalized interventions with diet, probiotics, and prebiotics.

Combining the terms of the green network, it is evident that the green cluster systematically classifies microbial communities with genetic and metagenomics techniques to understand the microbiome’s diversity. Moreover, the occurrences of the group’s terms in the word clouds and trend topic results confirm this transition. This also highlights the value of sequencing methods, which appeared in recent years of research evolution, where genetic identification is gaining more ground. The bibliometric analysis showed that the research focus is moving from classical microbiology to applying genetic methods. Studies from the Greek research community have demonstrated that these techniques (metagenomics and metaproteomic) can help identify additional microbial biomarkers for the personalized treatment of diseases such as cancer [70,71].

The terms *Firmicutes*, *Proteobacteria*, *Bacteroidetes*, and *Lactobacillus* indicate the investigation of microbial composition and different species in ecosystems or human samples. This highlights the value of new applications and high-throughput sequencing techniques in developing personalized medicine and nutritional analysis [72,73,74]. The terms nonhuman, animal experiment, and controlled study suggest that a significant part of the research focuses on experimental animal models [75,76,77,78].

The results of the subject areas, the bins of genetics and molecular biology, biotechnology, and microbiology, verify the interdisciplinary approach to microbiome analysis. The findings of this research production directly contribute to the creation of tools for understanding the composition of the microbiome and its genetic characteristics.

The composition and metabolic properties of microbes and the influence of the environment and the host constitute critical factors for shaping the structure of the microbiome [79,80]. Genetic and environmental factors interact to leverage the development of microorganisms. Objectives of experimental research in animal models concern the examination of the formation of populations of microbial phyla such as *Firmicutes*, *Proteobacteria*, and *Bacteroidetes.* These studies provide essential data on the structure of the microbiome and its differences depending on the environment, the host, and external conditions.

In addition, our understanding of the microbiome is evolving as bioinformatics tools are evolving. Mapping the microbiome, concerning human health, environmental processes, and ecosystem functions, provides valuable insights and constitutes a solid scientific background for microbial diversity by analyzing their molecular properties and features [81,82,83]. The aim is to enrich it with new knowledge, link it to clinical, nutritional, and environmental applications, and concurrently confirm the role of the microbiome in modern medical research, clinical microbiology, biotechnology, and the food industry.

The blue cluster focuses on the microbiome’s demographic, nutritional, and metabolic aspects. Variations in the microbiome between genders, age groups, and dietary patterns make research in this area crucial for developing personalized prevention and therapeutic strategies. Regarding the microbiome’s influence between genders, the differentiation in its composition and metabolic profile may also be linked to biochemical processes influenced by the hormones of the organisms in the two sexes [84,85].

The temporal evolution of publications also confirms this. In recent years, research on the microbiome has increasingly focused on its differentiation according to age, gender, and metabolic status. In particular, its connection with the red cluster shows that metabolic markers and nutrition are crucial for regulating the intestinal microbiota and preventing inflammatory diseases. At the same time, diets rich in plant fibers are associated with better metabolic health [86]. They reduce the level of oxidative stress in the body, improving inflammatory markers and health [87,88].

In addition, the connection with the green complex suggests that molecular techniques are used to investigate the effect of nutrition on the microbiome. The articles that stood out show that the microbiome’s diversity differs significantly between individuals of different ages, with older adults presenting reduced microbial diversity and an increased presence of potentially pathogenic species. The transition from normal to dysfunctional ageing passes through nutrition and its translation at the level of biological markers, mainly in middle age, where the nutritional profile significantly determines metabolic processes [89,90,91,92]. Specific metabolic abnormalities and microbial populations also characterize the metabolic pathways detected in obese individuals [93,94].

The reduction in biodiversity, the change in composition, and the dietary pattern differentiate the microbiome during the organism’s ageing and the change in metabolic indicators [95]. Different ages and genders are associated in various ways with different dietary interventions, which indicates the importance of personalized dietary and nutritional planning [95,96,97,98].

At the same time, racial differentiation is recorded, as studies show that the hormonal profile of men and women can influence the composition of the microbiome, influencing metabolic and immunological mechanisms. Particular emphasis is given to the microbiome’s relationship with nutrition, where data show that specific dietary patterns, such as the Mediterranean diet, are associated with increased microbial diversity and beneficial metabolic profiles [91,99,100]. In conclusion, the microbiome’s identity may also indicate the identity of ageing as a metabolic marker that connects different nutritional modifications, solar, and phylogenetic changes, and it will also constitute an innovative field of research in the future [100].

Beyond the descriptive bibliometric mapping, our findings provide key insights into emerging trends and research gaps. Despite the increase in microbiome-related publications in Greece and their potential to analyze microbiome composition, some research areas are still limited, such as the microbiome and neurodegenerative diseases and the effects of the microbiome on mental health disorders, personalized microbiome-based therapies, and research on the environment and the soil microbiome, since Greece has a strong tradition in the agri-food sector, research on environmental microbiomes, bioremediation, and interactions of soil microflora with crops remains relatively unexplored. Expanding research through targeted national funding in these directions would strengthen Greece’s contribution to cutting-edge microbiome science and ensure alignment with global research priorities.

No matter how extensive, bibliometric analysis has limitations, such as depth and analytical adequacy. Thus, for this particular bibliometric analysis, its limitations, without invalidating the adequacy and originality of the research, are the choice of a single database. Moreover, data from doctoral theses that have not yet been published could be incorporated into the research output of Greek scientists. It would make sense to examine individual scientific fields in isolation concerning the results of this analysis, which can be used as a starting point. Lastly, although the search language was English, data in the Greek language from other bibliographic sources were not utilized.

While Scopus is comprehensive and user-friendly for bibliometric analysis, limiting our search to one source excludes some potentially relevant research, including documents from other databases or languages beyond English. As a result, specific topics or contributions may be underrepresented. Nevertheless, this work is foundational for documenting microbiome research in Greece. Future endeavors can broaden the scope by integrating multiple bibliographic databases (e.g., Web of Science, PubMed) and non-English sources, reducing possible biases and capturing a more inclusive field snapshot. Overall, subsequent studies will likely refine and extend our outcomes, further elucidating how Greek scientists contribute to the evolving landscape of microbiome research.

## 5. Conclusions

This bibliometric analysis highlighted the dynamics of the Greek scientific community on the microbiome. In this vein, from 2018 onwards, a rapid increase in research production has been observed, and the microbiome’s linkage with human health and metabolic and autoimmune pathological conditions is emphatically underlined. The contribution of universities and institutions also includes microbiome research at the level of metagenomics, analysis, and the transition from the basic description of the microbiome to the search for clinical and therapeutic applications.

In addition, research involving Greek scientists is published in journals of high prestige and impact. The present study also highlights the importance of interdisciplinary approaches to studying the microbiome, such as medicine, biology, genetics, microbiology, and molecular biology.

In conclusion, the consistently increasing research output shows the prospects of Greek researchers for further research and innovation. Providing additional financial tools will open up new research horizons to produce high-level research on the microbiome and helps in deciphering its role in human health and many biotic processes.

## Figures and Tables

**Table 1:** Microbiome definitions and conceptual framework (adapted from [2]).

Conceptual Framework	Definition
Ecology	An ecological network defined by characteristic microbial communities occupying a specific environment with specific physicochemical properties. Includes not only microorganisms but also their activities concerning the environment. Microbial communities that include symbiotic and pathogenic microorganisms within an environment or a space in the body.
Organism–host interactions	A community of microorganisms (bacteria, fungi, and viruses) that inhabit a specific environment, particularly the microbial communities that live in or on the human body. The human microbiome includes the microorganisms living in the human body. These microbial communities are composed of a variety of eukaryotes, archaea, bacteria, and viruses.
Genomic methods	The entire genome of a microbiota. The collective genomes of microorganisms inhabiting a particular environment, particularly the human body. The microbiome includes all the genetic material within a particular location, such as the human gut. This can also be referred to as the microbiota metagenome. Microbiome is a term that describes the genome of all microorganisms, both symbiotic and pathogenic, that live in and on all vertebrates. The gut microbiome consists of the collective genome of microbes that inhabit the gut, including bacteria, archaea, viruses, and fungi. Microbiome: the genes and genome of the microflora, as well as the products of the microflora with the host environment.
Combinative definitions	Microbiome is the ecological community of symbiotic and pathogenic microorganisms that literally inhabit our bodies. Microbiome is the summation of microbiota and their genome in a specific environment. The genes and genome of the microflora, the products of the microflora, and the host environment.

**Table 2:** Five most influential papers according to citation count.

Title	Authors	Source	Year	Citations
Management of Helicobacter pylori infection-the Maastricht V/Florence consensus report	Malfertheier P. et al.	Gut, 66(1), pp. 6–30	2017	2338
Microbiome definition re-visited: old concepts and new challenges	Berg G. et al.	Microbiome, 8(1), 103	2020	1263
Obesity and cancer risk: Emerging biological mechanisms and perspectives	Avgerinos K.I. et al.	Metabolism: Clinical and Experimental, 92, pp. 121–135	2019	921
Agriculture and food animals as a source of antimicrobial-resistant bacteria	Economou V. and Gousia P.	Infection and Drug Resistance, 8, pp. 49–61	2015	549
Gut-Microbiota-Brain Axis and Its Effect on Neuropsychiatric Disorders with Suspected Immune Dysregulation	Petra A.I. et al.	Clinical Therapeutics, 37(5), pp. 984–995	2015	467

**Table 3:** VOS viewer clusters of “microbiome”.

Cluster Identification	Keywords
Red	human, intestine flora, review, gastrointestinal microbiome, microbiology, animals, metabolism, dysbiosis, probiotic agent, priority journal, probiotics, physiology, inflammation, obesity, gut microbiota, risk factor, immunology, immune response, pathogenesis, prebiotic agent, inflammatory bowel disease, pathophysiology, antibiotic agent, drug effect, pathology, gut microbiome, unclassified drug
Green	nonhuman, microbiome, article, microbiota, controlled study, microflora, genetics, microbial community, rna 16s, bacteria, microbial diversity, metagenomics, Firmicutes, Proteobacteria, high-throughput sequencing, lactobacillus, animal experiment, polymerase chain reaction, Bacteroides, DNA extraction
Blue	female, male, adult, biological marker, diet, middle-aged, metabolomics, aged

## Data Availability

The original contributions presented in this study are included in the article. Further inquiries can be directed to the corresponding author.
